# Evaluating multimodal physiological signals for fear detection: relative utility of pupillometry, heart rate, and EEG

**DOI:** 10.3389/fnhum.2025.1605577

**Published:** 2025-07-29

**Authors:** Yuki Ebato, Tomita Saki, Isshu Wakita, Ayumu Ueno, Tomoaki Ishibashi, Tetsuya Takahashi, Sou Nobukawa

**Affiliations:** ^1^Department of Computer Science, Chiba Institute of Technology, Narashino, Japan; ^2^Graduate School of Information and Computer Science, Chiba Institute of Technology, Narashino, Japan; ^3^Department of Neuropsychiatry, University of Fukui, Fukui, Japan; ^4^Research Center for Child Mental Development, Kanazawa University, Kanazawa, Japan; ^5^Uozu Shinkei Sanatorium, Uozu, Japan; ^6^Research Center for Mathematical Engineering, Chiba Institute of Technology, Narashino, Japan; ^7^Department of Preventive Intervention for Psychiatric Disorders, National Center of Neurology and Psychiatry, National Institute of Mental Health, Tokyo, Japan

**Keywords:** electroencephalography, fear monitoring, multimodal measurement, pupil dynamics, heart rate

## Abstract

**Introduction:**

Fear is a fundamental emotion essential for survival; however, excessive fear can lead to anxiety disorders and other adverse consequences. Monitoring fear states is crucial for timely intervention and improved mental well-being. Although functional magnetic resonance imaging (fMRI) has provided valuable insights into the neural networks associated with fear, its high cost and environmental constraints limit its practical application in daily life. Electroencephalography (EEG) offers a more accessible alternative but struggles to capture deep brain activity. Physiological measures such as pupil dynamics and heart rate can provide indirect insights into these deeper processes, yet they are often studied in isolation. In this context, we aimed to evaluate the practical effectiveness and limitations of a multimodal approach that combines pupil dynamics and heart rate—indirect indicators of deep brain activity—with EEG, a temporally precise but spatially limited measure of cortical responses.

**Methods:**

We simultaneously recorded EEG, pupillometry, and heart rate in 40 healthy male participants exposed to fear-inducing and neutral visual stimuli, while also assessing their psychological states.

**Results:**

Fear-inducing stimuli elicited distinct physiological responses, including increased occipital theta power, pupil dilation, and decreased heart rate. Notably, pupil size was the most sensitive discriminator of emotional state, though the integration of modalities yielded only limited improvement in classification accuracy.

**Discussion:**

These findings provide empirical support for the feasibility of multimodal physiological monitoring of fear and underscore the need for further refinement for real-world applications.

## 1 Introduction

Fear is a fundamental emotion triggered by realistic and severe threats [reviewed in Blanchard and Blanchard (2008); Davis et al. (2010); Grupe and Nitschke (2013); Malezieux et al. (2023)]. Its neural basis involves the transmission of environmental information from primary and associative sensory cortices and the thalamus to the amygdala, where this information is evaluated for threat detection. When a threat is detected, the outcome of this evaluation triggers behavioral and physiological responses through connections to the hypothalamus and brainstem. Additionally, the amygdala projects to extensive cortical regions and the hippocampus, adjusting cognitive functions such as attention, memory, and decision-making. Thus, fear plays a vital role in survival by facilitating rapid responses and enhancing learning and memory (Phelps and LeDoux, 2005; LeDoux, 2013). However, excessive fear can negatively affect the body and lead to anxiety disorders, phobias, avoidance behaviors, and activity limitations [reviewed in Pittig et al. (2018)]. Therefore, accurately and efficiently monitoring an individual's fear state—especially through non-invasive, real-time techniques—is essential for enabling appropriate interventions and adaptive support in daily life. Multimodal physiological monitoring, combining neural and autonomic indicators, holds promise as a practical approach for this purpose (Sheikh et al., 2021; Williams and Pykett, 2022).

One method of monitoring fear involves assessing the activity of neural networks centered on the amygdala, a deep brain structure involved in fear evaluation [reviewed in Domínguez-Borràs and Vuilleumier (2022)]. Over the past few decades, functional magnetic resonance imaging (fMRI) studies have revealed the characteristics of these neural networks, particularly the interactions between the amygdala and the cerebral cortex, hippocampus, thalamus, and hypothalamus (Richardson et al., 2004; Kral et al., 2024). However, this approach is limited by high costs and environmental constraints, making it challenging to monitor mental states in everyday settings. Alternatively, electroencephalography (EEG)-based approaches are more cost-effective and limited by fewer environmental constraints. Although EEG has inherent limitations in spatial resolution and direct access to deep brain structures, its high temporal resolution and portability make it a promising tool for monitoring cortical responses related to emotional states in real-world settings (Chien et al., 2017; Sperl et al., 2019; Chen et al., 2021). Recent advancements in wearable EEG technology have spurred increasing interest in their application for monitoring mental states in real-world environments (Anders and Arnrich, 2022).

In addition to EEG, other physiological data, such as pupil dilation and heart rate, serve as powerful tools for monitoring mental states related the neural activities of amygdala and locus coeruleus (LC), a central hub for the noradrenergic (NA) [reviewed in Sheikh et al. (2021); Johansson and Balkenius (2018)]. Through these neural activities, emotions, such as fear, influence both behavioral and physiological responses. Furthermore, these physiological responses reflect activity in deep brain regions that cannot be captured by EEG alone. Therefore, combining EEG with behavioral and physiological data in a multimodal approach can complement the spatiotemporal coverage of each modality, enhancing the accuracy of mental state estimation (Anders and Arnrich, 2022). Although this approach offers potential advantages, multimodal strategies for monitoring mental states remain underexplored, as most studies focus on either EEG alone (Chien et al., 2017; Sperl et al., 2019; Anders and Arnrich, 2022) or single-modality approaches using behavioral or physiological data (Leuchs et al., 2017; Rafique et al., 2020).

In this context, we aimed to evaluate the practical effectiveness and limitations of a multimodal approach that combines pupil dynamics and heart rate—which are considered indirect indicators of deep brain activity—with EEG, a temporally precise but spatially limited measure of cortical activity. Rather than seeking to uncover novel neural mechanisms, the present study focuses on validating whether integrating these complementary modalities improves the accuracy of emotional state detection, particularly for fear. To this end, we conducted simultaneous EEG, pupillometry, and heart rate measurements in 40 male participants while presenting fear-inducing and neutral visual stimuli. The participants were also assessed for trait and state anxiety, enabling us to evaluate multimodal indicators in relation to individual emotional characteristics. By evaluating the relative utility and limitations of each physiological modality, this study seeks to contribute to the development of practical, real-world emotional monitoring systems. Our results revealed that pupil dilation following the stimuli was the most sensitive indicator distinguishing between fear and neutral conditions. However, integrating multiple modalities yielded limited classification improvements. These findings offer empirical insights into the relative utility of each physiological signal and suggest future directions for real-world emotional state monitoring.

## 2 Materials and methods

### 2.1 Participants

Forty male participants (aged 18–25 years) were recruited from the student body at Chiba Institute of Technology for this study. None of the participants had neurological impairments or behavioral difficulties, and all had normal vision or vision corrected to normal with contact lenses. Here, based on an a priori power analysis assuming a medium effect size [partial η^2^ = 0.06; (Cohen, 2013)], statistical power of 0.80, and two repeated measures, the minimum required sample size was estimated to be 33 participants. Female participants were excluded to avoid the potential influence of menstrual-related psychological stress on the state anxiety test. Participants were instructed to refrain from consuming caffeinated or alcoholic beverages and engaging in strenuous exercise on the day before and the day of the measurements. Written informed consent was obtained prior to participation. The Ethics Committee of Chiba Institute of Technology approved all experimental protocols and methods for this study (approval number: 2023-05-01). All procedures were conducted in accordance with the principles outlined in the Declaration of Helsinki.

Table 1 presents the data for all participants. In this study, the Japanese version of the State-Trait Anxiety Inventory (STAI), developed by Spielberger and colleagues, was used to measure participants' anxiety levels (Spielberger et al., 1971). The STAI is widely recognized for its reliability and validity and is frequently used in educational and clinical settings. The STAI effectively captures trait anxiety, reflecting an individual's general tendency to experience anxiety and state anxiety, which varies according to situational factors, providing a comprehensive measure of psychological state. In this study, the STAI was employed to assess participants' baseline anxiety levels. The STAI evaluates anxiety across two distinct dimensions: state anxiety (A-State) and trait anxiety (A-Trait). State anxiety (A-State) assesses participants' transient feelings of anxiety, while trait anxiety (A-Trait) indicates their general tendency toward anxiety over time. Each anxiety dimension consists of 20 items. Participants rated their own psychological state on a 4-point Likert scale, ranging from 1 (rarely) to 4 (almost always). Scores for each item were summed, resulting in a total score for each dimension, with possible scores ranging from 20 to 80. Higher scores indicate higher levels of anxiety. The STAI was administered before the visual stimulus task to assess baseline anxiety. The STAI scores were calculated by aggregating participants' responses for each item, resulting in total scores for A-State and A-Trait.

**Table 1:** Demographic characteristics of all participants are represented as means (range, standard deviation [SD]).

**Number of participants**	***N* = 40**
Age (years)	20.575 (18-25, 1.693)
Sex (male/female)	40/0
A-state	40.375 (23–55, 6.758)
A-trait	45.075 (23–60, 10.307)

The STAI was administered before the visual stimulus task and its scores were composed of scores for A-state and A-trait.

### 2.2 Experimental procedures

**Figure 1:**
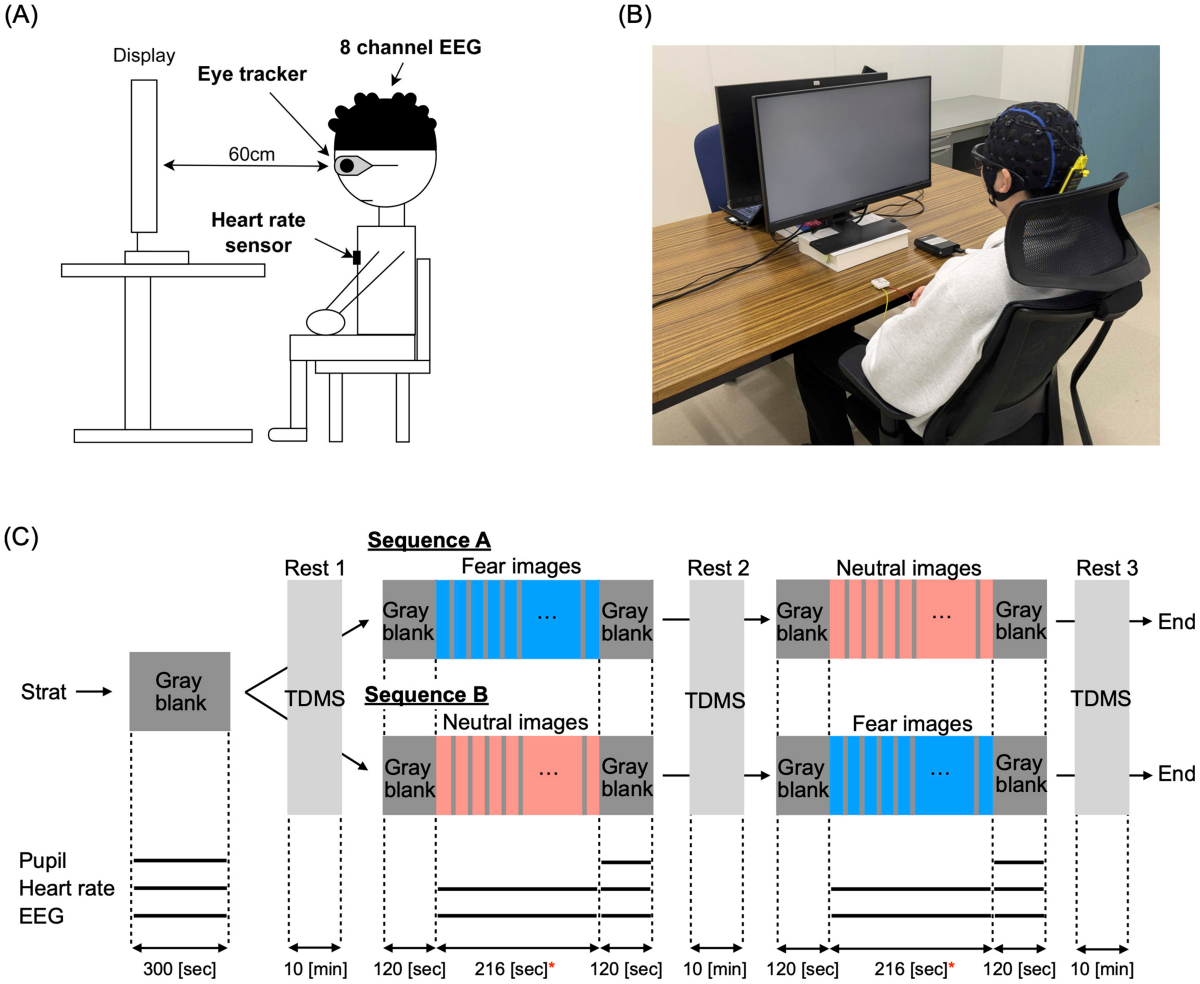
**(A)** Overview of the experimental setup. *Indicates significance at *p* < 0.05. Measurement equipment included a glasses-type eye tracker (Tobii Pro Glasses 3, Tobii Technology K.K.), an 8-channel wearable electroencephalography (EEG) device (NB1-EEG, East Medic Corporation), and a wearable heart rate monitor (my Beat, WHS-1). An LCD monitor was used for visual stimulus presentation. **(B)** Measurement scene during the presentation of a gray blank image. **(C)** Schedule for presenting visual stimuli, including gray blank images, fear images, and neutral images. Fear and neutral images were presented for 10 sec, followed by a 2-s gray blank image. During each 10-min resting period, participant's monetary mood states were evaluated using the Two-Dimensional Mood Scale (TDMS). The black solid lines at the bottom represent the evaluation periods for pupil diameter, heart rate, and EEG measurements.

Figure 1A presents an overview of the experimental setup. The measurement equipment included a glasses-type eye tracker (Tobii Pro Glasses 3, Tobii Technology K.K.), an 8-channel wearable EEG device (NB1-EEG, East Medic Corporation), and a wearable heart rate monitor (my Beat, WHS-1, UNION TOOL CO.). Participants sat in a chair with a backrest while wearing the eye tracker, EEG device, and heart rate monitor during the experiments. An LCD monitor with a vertical size of 597.6 cm (1920 pixels) and a horizontal size of 336.2 cm (1,080 pixels) was used for visual stimulus presentation. The effective field of view for the participants was ±26.47° horizontally and ±6.48° vertically. The experiment was conducted in a laboratory with an illuminance range of 300 lx to 600 lx, measured using a lux meter compliant with JIS C 1609-1:2006 Class A linear type. In Figure 1B, the measurement scene is shown while presenting a gray blank image.

For the visual stimulation task, we selected 36 images from the International Affective Picture System (IAPS) based on each image's scores (mean pleasure/arousal), consisting of 18 neutral images (mean pleasure/arousal = 4.5, 6.2) and 18 fear images (mean pleasure/arousal = 2.1, 6.6). The fear images included depictions of weapons, corpses, and murder scenes, while the neutral images featured items like snakes, spiders, and sharks. Additionally, we prepared gray (neutral) images that only displayed a gray blank image with *R* = 0, *G* = 0, *B* = 0 in the RGB scale. In the experiment, to balance the presentation order of the images, participants were randomly divided into two groups: group sequence-A (22 participants) and group sequence-B (18 participants) (see demographic characteristics of both groups in Table 2). As demonstrated in Figure 1C, group sequence-A was first shown a gray blank image for 5 min, followed by a 10-min break, and then shown fear images for 216 s. After another 10-min break, participants were shown neutral images for 216 s, followed by a final 10-min break. Group sequence B was first shown gray images for 5 min, followed by a 10-min break, then neutral images for 216 s, another 10-min break, and finally fear images for 216 s, ending with a 10-min break. Additionally, gray images were presented for 2 min before and after each presentation of fear and neutral images. During each presentation, participants viewed each image for 10 s, followed by a 2-s gray image, and then another 10-s display, repeating this cycle for a total of 216 s. A 10-min resting period was given before and after the visual stimulation task to ensure participants had ample rest.

**Table 2:** Demographic characteristics of sequence-A and sequence-B participants are represented as means (range, standard deviation [SD]).

**Evaluation indexes**	**Group sequence-A**	**Group sequence-B**	***p*-value**
Number of participants	*N* = 22	*N* = 18	—
Age (years)	20.545 (18-23, 1.535)	20.611 (18–25, 1.914)	0.905
Sex (male/female)	22/0	18/0	—
A-state	39.000 (23–55, 7.584)	42.056 (31-52, 5.319)	0.157
A-trait	43.955 (23–60, 11.303)	46.444 (26-59, 9.070)	0.454

There are no significant differences in age, sex ratio, A-state, and A-trains between groups of sequence A and sequence B.

Furthermore, to evaluate participants' momentary mood states during each resting period, we used the Two-Dimensional Mood Scale (TDMS) (Sakairi et al., 2013). The TDMS is a psychological assessment tool developed for self-monitoring and self-regulation, assessing mood states based on two dimensions: pleasure and arousal. This scale allows participants to self-assess their mood using eight adjectives, such as “energized” and “relaxed,” rated on a 6-point Likert scale (0: not at all, 5: very much). Each mood state is classified into one of the following four quadrants based on the combination of pleasure and arousal: high-energy pleasure (e.g., lively), high-energy discomfort (e.g., tense), low-energy pleasure (e.g., calm), and low-energy discomfort (e.g., lethargic). This approach visually represents momentary psychological states, enabling a quantitative understanding of mood changes. In this study, TDMS was administered at three time points: before the task, after the first image presentation, and after the second image presentation. To evaluate mood fluctuations, difference scores were calculated by subtracting the pre-task baseline from each post-task score. This approach allowed us to capture short-term, task-induced changes in pleasure and arousal in response to specific psychological stimuli. For example, a decrease in energy levels and a decline in emotional stability following exposure to fear-inducing stimuli may indicate a strong psychological impact.

### 2.3 Recording

#### 2.3.1 Pupil size

Pupil diameter was measured using the glasses-type eye tracker (Tobii Pro Glasses 3) with a sampling frequency of 100 Hz. For segments missing due to blinking, linear interpolation was performed, treating the range, including 0.1 s before and after the missing segment, as missing values. Furthermore, all segments of the pupil responses for the gray blank images, fear images, and neutral images in any participant that deviated by more than three SDs from that participant's mean for the corresponding condition were treated as missing values, and linear interpolation was performed accordingly. Finally, a 10 Hz low-pass filter was applied. The average pupil diameter during the 5-min presentation of a gray blank image was used as the baseline for each participant. The pupil size was then calculated as the difference between this baseline and the time-averaged pupil diameter during the 3-min presentation of a gray blank image following the visual stimulus task.

#### 2.3.2 Heart rate

The heart rate measured by the wearable heart rate sensor (my Beat, WHS-1/RRD-1) was used to calculate the average R-R interval (RRI) during a 5-min presentation of a gray blank image, which served as the baseline for each participant. The difference from this baseline was then calculated for the average RRI during the visual stimulus tasks with fear and neutral image groups, as well as during the gray blank image presentation following the visual stimulus tasks.

#### 2.3.3 Electroencephalography

EEG data were recorded using a wearable 8-electrode EEG device (NB1-EEG) with a sampling rate of 121.37 Hz. Using EEGLAB 2023.1, the data from each segment, consisting of a 5-min gray blank image, the visual stimulation task, and a gray blank image after the task, were resampled to 120 Hz and filtered with a 1-40 Hz band-pass filter. Independent Component Analysis (ICA) was then performed on each segment to classify each component into Brain, Muscle, Eye, Heart, or Other, according to the highest probability. Components classified as categories other than the Brain were removed. For the analysis, electrodes from the frontal (F3, F4) and parieto-occipital (PO3, PO4) regions were selected because these sites have been reliably associated with emotional and visual processing (Keil et al., 2002; Hajcak et al., 2010). The relative powers of the theta, alpha, and beta waves were calculated for each electrode, and averaged within the frontal and occipital regions.

#### 2.3.4 Simultaneous measurements

The start timing of pupil size and EEG measurements was controlled using Python, and timestamps of visual stimuli during the measurements were also recorded. For the heart rate monitor, the start switch was pressed within 1 s before or after the start of pupil diameter and EEG measurements.

### 2.4 Evaluation index

The evaluation indices for pupil size, heart rate, and EEG were calculated using the average of the left and right pupil diameters, the average RRI value, and the relative power from the frontal (F3, F4) and occipital (PO3, PO4) electrodes, respectively. For EEG, the relative power of theta (2–4 Hz), alpha (8–13 Hz), and beta (13–30 Hz) bands was calculated for each electrode and averaged for the frontal and occipital regions. For pupil size, deviation from baseline was determined by subtracting the baseline value from the time-averaged left and right pupil diameters during the presentation of the gray blank image following the visual stimulation task. During the visual stimulation task, image luminance was not constantly maintained to ensure a vivid and naturalistic presentation. As a result, pupil diameter was affected not only by arousal and emotional valence, but also by luminance fluctuations. To minimize this confounding influence, we excluded the stimulation period from the pupil-based evaluation. In contrast, RRI values and EEG relative power were calculated by averaging data from both the visual stimulation period and the subsequent gray blank image.

### 2.5 Statistical analysis

The difference between neutral- and fear-induced responses was evaluated using repeated measures analysis of variance (ANOVA) for crossover trial assessment. The between-subject factor was sequence (sequence-A and sequence-B), while the within-subject factors were image type (neutral or fear) and presentation period. Each response was evaluated based on the change from the baseline value measured during the initial gray image presentation. Statistical significance was set at *p* < 0.05 using the Greenhouse-Geisser correction for both psychological responses (arousal and comfort levels) and physiological responses (pupil size, RRI, EEG power). To control for the risk of Type I error due to multiple comparisons among physiological indices, Bonferroni correction was additionally applied.

We utilized the receiver operating characteristic (ROC) curve and its area under the curve (AUC) based on logistic regression to assess the ability of biosignals to distinguish which stimulus (fear-induced or neutral) was presented to participants. For cross-validation, leave-one-out cross-validation (LOOCV) was applied. A random classification corresponds to AUC = 0.5, while a perfect classification corresponds to AUC = 1.0.

All statistical analyses were conducted using IBM SPSS Statistics (Version 29.0.2) or MATLAB (Version R2023b).

## 3 Results

### 3.1 Psychological responses and their relation to trait and state anxiety

**Figure 2:**
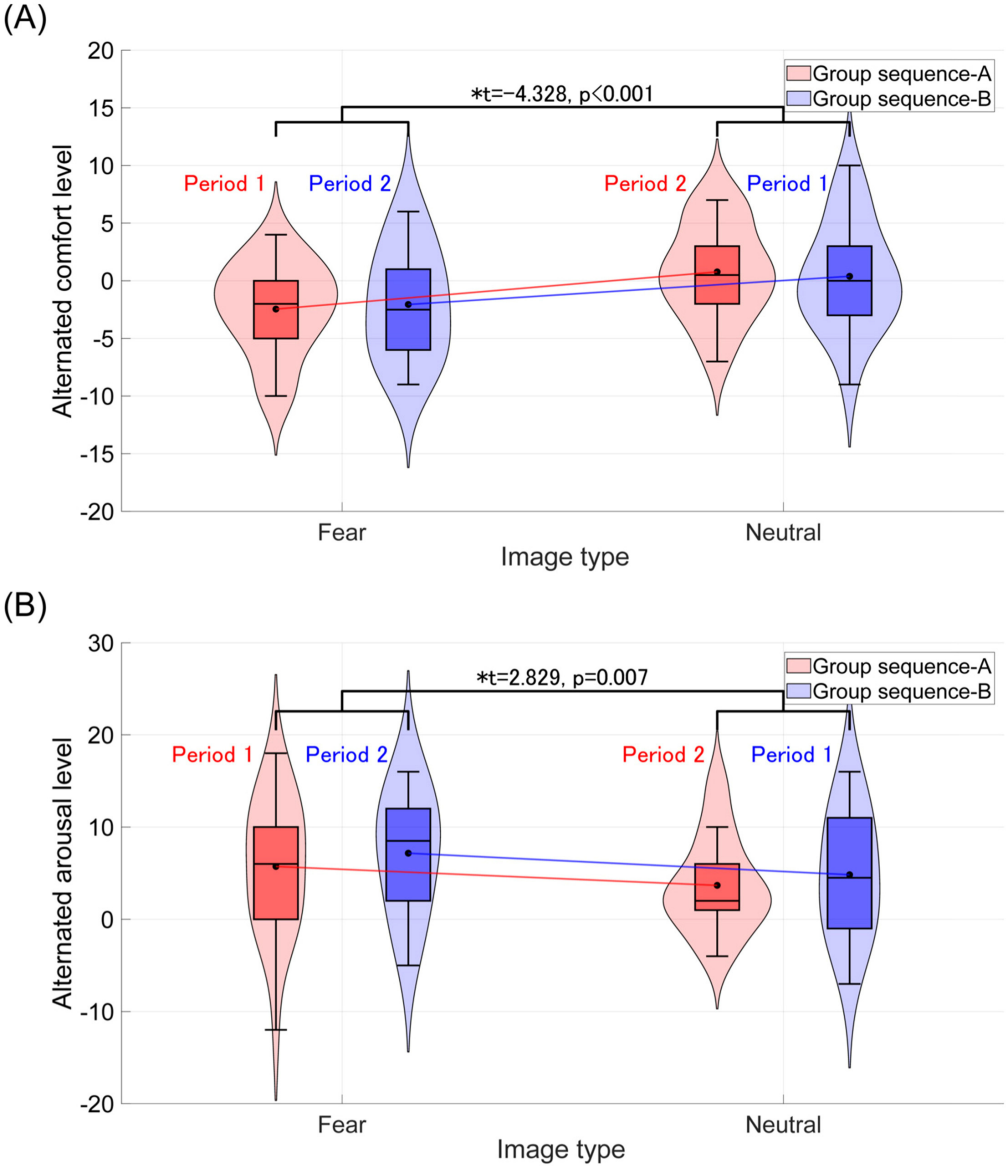
Violin plots and boxplots show the distribution of TDMS alternated scores after the stimuli (rest 2 and rest 3) compared to the beginning of the tasks (rest 1) in the sequence A and sequence B groups. *Indicates significance at *p* < 0.05. **(A)** Distribution of comfort levels. The average comfort level across groups (sequence A and sequence B) after representing the fear-inducing images decreases compared to that after the neutral stimulus. **(B)** Distribution of arousal levels. The average arousal level across groups (sequence A and sequence B) after representing the fear-inducing images increases compared to that after the neutral stimulus.

For each group, sequence A and sequence B, comfort and arousal levels following the visual stimulus presentation were measured using TDMS and evaluated through a crossover trial. The results of comfort and arousal evaluations are presented in Tables 3, 4, respectively. Both comfort and arousal levels showed a significant main effect related to image type (fear/neutral). Additionally, Figure 2 displays violin plots and boxplots showing the distribution of TDMS alternated scores after the stimuli compared to the baseline (beginning of the tasks) in the sequence A and sequence B groups. Subplots (A) and (B) correspond to the comfort level and arousal level, respectively. The presentation of fear-inducing images resulted in a significant decrease in comfort compared to neutral images. Conversely, fear-inducing images led to a significant increase in arousal compared to neutral images.

**Table 3:** Results of repeated-measures analysis of variance (ANOVA) for crossover trial on comfort levels with factors of image type, presentation sequence, and measurement period.

**Factors**	***F*-value (*p*-value, η^2^)**
Image type	17.736 (< 0.001, 0.318)
Sequence	0.000 (0.995, 0.000)
Period	0.338 (0.564, 0.009)

Significant results (*p* < 0.05) are shown in bold.

**Table 4:** Results of repeated-measures analysis of variance (ANOVA) for crossover trial on arousal levels with factors of image type, presentation sequence, and measurement period.

**Factors**	***F*-value (*p*-value, η^2^)**
Image type	7.832 (0.008, 0.171)
Sequence	0.496 (0.486, 0.013)
Period	0.034 (0.855, 0.001)

Significant results (*p* < 0.05) are shown in bold.

Moreover, correlations between A-state and A-trait in the STAI scores were analyzed. The results showed a significant and strong Pearson's correlation, *r* = 0.66 (*p* < 0.001). Regarding the relationship between alternated scores in the TDMS and the STAI scores, Table 5 presents the results of the correlation analysis, combining both groups for each type of image. A strong negative correlation was observed between the A-state and alternated arousal scores. This pattern suggests that participants with higher A-state scores tended to exhibit smaller changes in arousal, which is consistent with previous findings indicating reduced autonomic reactivity and restricted autonomic flexibility in high-anxiety individuals (Thayer and Lane, 2000; Friedman, 2007; Porges, 2007; Asbrand et al., 2022).

**Table 5:** Pearson's correlation *r*-value (*p*-value) between STAI scores and TDMS alternated scores based on the score after representing a gray blank image.

**Factors**	**A-state**	**A-trait**
Alternated comfort score by fear-image	0.242(0.133)	0.244(0.129)
Alternated arousal score by fear-image	**−0.361(0.022)**	−0.202(0.210)
Alternated comfort score by neutral-image	0.148(0.363)	0.034(0.835)
Alternated arousal score by neutral-image	−0.134(0.410)	0.068(0.678)

Significant results (*p* < 0.05) are shown in bold.

### 3.2 Physiological responses for pupil size, heart rate, and electroencephalography

**Figure 3:**
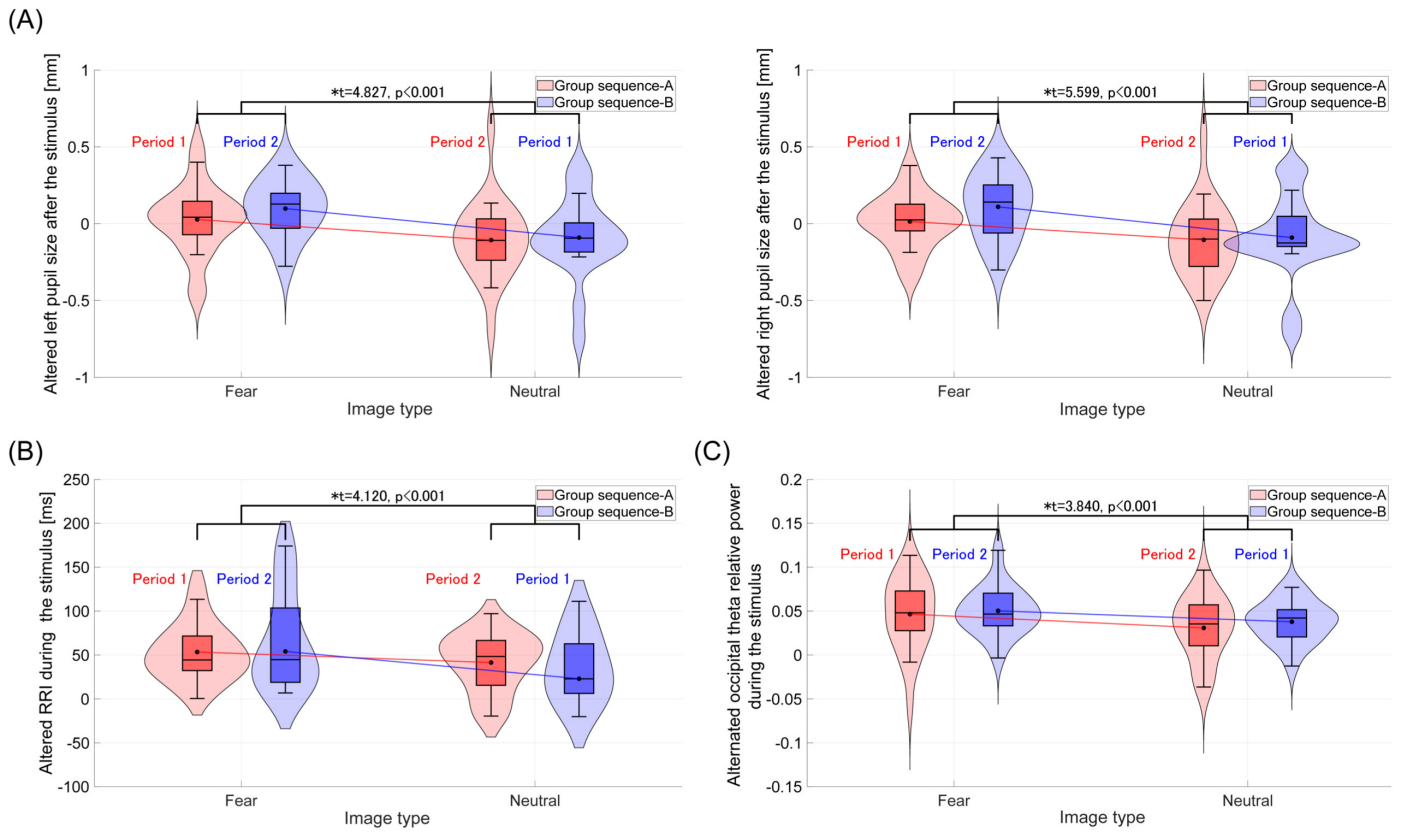
Violin plots and boxplots illustrate the distribution of alternated physiological responses after or during the stimuli, compared to the baseline (rest 1), in sequence A and B groups. *Indicates significance at *p* < 0.05. **(A)** After the stimulus, the distribution of alternated pupil sizes in the left and right eyes. The average alternated pupil sizes in both eyes across groups (sequence A and sequence B) significantly increase following fear-inducing images compared to neutral images. **(B)** Distribution of alternated RRI during the stimulus. Compared to neutral images, the average alternated RRI across groups significantly increases during fear-inducing images. **(C)** Distribution of alternated occipital theta relative power during the stimulus. The average occipital theta relative power across groups significantly increases during fear-inducing images compared to neutral images.

For each group, sequences A and B, the alternated pupil size, RRI, and frontal/occipital relative powers during and/or after the visual stimulus presentation were evaluated in a crossover trial, as shown in Table 6. Significant main effects of image type (fear vs. neutral) were observed in left and right pupil diameters following stimulus presentation, with corresponding Cohen's *f* values of *f* = 0.789 (left) and *f* = 0.939 (right), indicating large effect sizes. Similarly, significant effects were found for RRI and occipital theta relative power during stimulus presentation, with Cohen's *f* values of *f* = 0.720 and *f* = 0.606, respectively, also indicating large effect sizes. Figures 3A–C display violin plots and boxplots that illustrate the distribution of these alternated physiological responses. The results indicate that the average alternated values of these physiological responses across groups (sequence A and sequence B) significantly increased following fear-inducing images compared to neutral images.

**Table 6:** Results of repeated measures ANOVA for crossover trial regarding physiological responses for pupil size, heart rate, and EEG.

**Evaluation indexes**	**Factors**	***F*-value (*p*-value, η^2^) during stimulus**	***F*-value (*p*-value, η^2^) after stimulus**
Size of left pupil	Image type	—	23.659 (< 0.001, 0.384)
	Sequence	—	0.422 (0.520, 0.011)
	Period	—	0.664 (0.420, 0.017)
Size of right pupil	Image type	—	33.573 (< 0.001, 0.469)
	Sequence	—	0.697 (0.409, 0.018)
	Period	—	2.100 (0.155, 0.052)
RRI of heart rate	Image type	19.752 (< 0.001, 0.342)	0.068 (0.795, 0.002)
	Sequence	0.301 (0.586, 0.008)	0.036 (0.850, 0.001)
	Period	3.869 (0.057, 0.092)	0.644 (0.427, 0.017)
Frontal theta relative power	Image type	0.203 (0.655, 0.005)	0.656 (0.423, 0.017)
	Sequence	0.325 (0.572, 0.008)	0.042 (0.838, 0.001)
	Period	0.014 (0.907, 0.000)	2.288 (0.139, 0.057)
Occipital theta relative power	Image type	13.969 (< 0.001, 0.269)	0.013 (0.911, 0.000)
	Sequence	0.296 (0.589, 0.008)	0.203 (0.655, 0.005)
	Period	0.187 (0.668, 0.005)	0.589 (0.448, 0.015)
Frontal alpha relative power	Image type	0.384 (0.539, 0.010)	0.578 (0.452, 0.015)
	Sequence	0.522 (0.474, 0.014)	1.471 (0.233, 0.037)
	Period	0.375 (0.544, 0.010)	0.523 (0.474, 0.014)
Occipital alpha relative power	Image type	0.150 (0.701, 0.004)	0.686 (0.413, 0.018)
	Sequence	0.364 (0.550, 0.009)	0.023 (0.880, 0.001)
	Period	0.680 (0.415, 0.018)	0.463 (0.500, 0.012)
Frontal beta relative power	Image type	0.518 (0.476, 0.013)	2.083 (0.157, 0.052)
	Sequence	0.396 (0.533, 0.010)	1.604 (0.213, 0.040)
	Period	1.652 (0.206, 0.042)	0.000 (0.983, 0.000)
Occipital beta relative power	Image type	1.770 (0.191, 0.045)	0.725 (0.400, 0.019)
	Sequence	0.171 (0.682, 0.004)	0.729 (0.398, 0.019)
	Period	0.074 (0.786, 0.002)	0.752 (0.391, 0.019)

Significant results (*p* < 0.05) (Bonferroni-corrected) are shown in bold.

**Figure 4:**
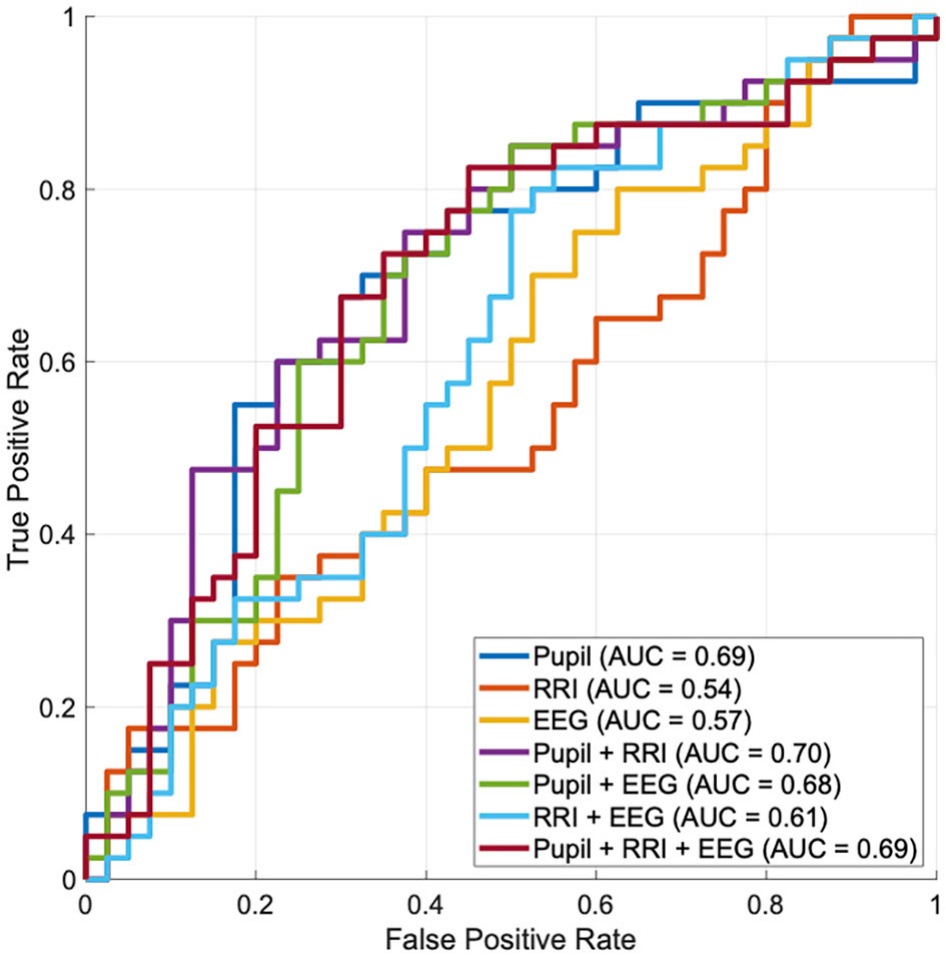
The ability to distinguish whether participants were presented with fear-induced or neutral stimuli was evaluated using the receiver operating characteristic (ROC) curve, based on pupil size after the stimulus, as well as the RRI and occipital theta relative power during the stimulus. Here, the right and left pupil sizes show a high correlation (Pearson's correlation *r* = 0.930); therefore, their values are averaged to obtain a single pupil size measure. Based on each ROC curve, the area under the curve (AUC) is shown in the legends.

Further, we evaluated the ability to distinguish which stimulus (fear-induced or neutral) was presented to participants, based on pupil size after the stimulus, as well as RRI and occipital theta relative power during the stimulus. Here, the right and left pupil sizes showed a high correlation (Pearson's correlation *r* = 0.930); therefore, their values were averaged to obtain a single pupil size measure. In Figure 4, the ROC curve and AUC values obtained using logistic regression are presented. As a result, pupil size demonstrated the highest ability to distinguish stimuli (AUC = 0.69) among the other unimodal biosignals. Regarding combinations of these biosignals, combining RRI with pupil size slightly improved the detection ability (AUC = 0.70).

## 4 Discussion

In this study, we aimed to investigate the neural activity associated with fear-induced cortical and deep brain responses, as reflected in heart rate and pupil changes. We simultaneously measured EEG, pupillometry, and heart rate in participants exposed to fear-inducing and neutral visual stimuli alongside assessments of their psychological responses. The results showed that fear-inducing stimuli decreased comfort levels and increased arousal, and these changes correlated more strongly with state anxiety than with trait anxiety. Furthermore, fear-inducing stimuli led to increased pupil size after the stimulus, a rise in RRI during the stimulus, and occipital theta band power during the stimulus.

### 4.1 Psychological responses

We observed that viewing the fear-inducing images lowered pleasantness and increased arousal, with a strong positive correlation between trait and state anxiety. This result is consistent with prior findings that an individual's enduring predisposition toward anxiety (trait anxiety) strongly influences their short-term anxiety in specific situations (state anxiety) (Leal et al., 2017). Regarding the effect of trait anxiety on responses to neutral- and fear-inducing images, no significant correlation was observed in either comfort or arousal. These results suggest that trait anxiety may not strongly influence physiological arousal responses to intense fear stimuli. In contrast, individuals with higher state anxiety exhibited lower changes in arousal (see Table 5). As noted in the Methods section (Section 2.2), the fear-inducing images were selected based on normative ratings from IAPS, with low valence (mean = 2.1) and high arousal (mean = 6.6), indicating strong emotional unpleasantness and intensity. Given the high baseline arousal induced by these stimuli, it is possible that their effects were already near ceiling levels, thus reducing observable modulation by individual differences in trait anxiety.

### 4.2 Physiological responses

#### 4.2.1 Individual response

We discuss the reasons underlying the observed individual physiological responses. First, regarding the increase in RRI during the presentation of fear-inducing images, previous studies have reported a decrease in heart rate due to vagal activation triggered by fear stimuli (Bradley et al., 2008; Hermans et al., 2013). Our findings are consistent with those of earlier studies. Second, concerning the increase in pupil size following the presentation of fear-inducing stimuli, it is notable that the LC, the origin of the neural pathway controlling pupil size, strongly influences pupil dynamics via the NA system (Aston-Jones and Cohen, 2005). The heightened arousal induced by fear stimuli likely caused pupil dilation, which aligns with the psychological response of increased arousal measured using the TDMS (see Figure 2). Finally, the enhancement of occipital theta power during fear-inducing stimuli can be explained by the following mechanism: when the amygdala evaluates a stimulus as a threat, it provides attentional connections to the basal forebrain and various cortical areas (Pinto et al., 2013; Peck and Salzman, 2014; Chaves-Coira et al., 2018), leading to increased theta power in the temporal and occipital regions (Torrence et al., 2021). Our results align with this mechanism, showing enhanced occipital theta power in response to fear-inducing stimuli.

We discuss why pupil dilation exhibited the highest ability to distinguish between fear-induced and neutral stimuli, compared to RRI and occipital theta relative power. First, pupil size reflects neural activity in deep brain regions related to fear and arousal, such as the LC and amygdala (Johansson and Balkenius, 2018), which cannot be detected by EEG signals. Furthermore, the LC is directly involved in the neural pathway controlling pupil diameter, serving as its primary source. Therefore, pupil dilation more accurately reflects fear and arousal compared to other physiological signals.

It should be noted that, unlike EEG and RRI, pupil dilation was analyzed only during the post-stimulus blank period. This decision was made because the luminance of the fear-inducing and neutral images varied in order to maintain naturalistic and vivid presentation, which can significantly influence pupil size. Although some studies have attempted to correct for luminance-related effects using regression-based models (Hermans et al., 2013), such methods require careful calibration and validation, especially in dynamic visual environments. In this initial study, we prioritized isolating emotionally driven pupil responses by excluding the stimulation period altogether. Nevertheless, future work should consider incorporating luminance correction models or presenting stimuli with controlled brightness to enable more direct cross-modal comparisons of temporal dynamics.

One limitation of this study is the assumption that all images categorized as “neutral” or “fear-inducing” would elicit consistent emotional responses across participants. In particular, certain stimuli considered neutral (e.g., snakes or spiders) may evoke fear in some individuals, while others may not perceive fear-inducing images as strongly aversive. This variability in subjective perception could have influenced the distribution of behavioral responses, as reflected in the skewness observed in Figure 3. Future studies should include individual-level ratings of emotional valence for each image to better account for such inter-individual differences. Another limitation concerns the analysis of hemispheric asymmetries in neural and physiological signals. While we focused on averaged activity across hemispheres in the current study, previous research suggests that emotional processing may involve lateralized neural responses (Harmon-Jones and Gable, 2018). A thorough investigation of hemispheric effects would also require examining potential lateralization in other modalities such as pupil dynamics, which may be influenced by lateralized neural circuits such as the bilateral LCs (Samuels and Szabadi, 2008; Liu et al., 2017; Nobukawa et al., 2021; Kumano et al., 2022). However, clarifying these possibilities requires comprehensive multimodal analyses, such as examining hemispheric asymmetries in neural activity and pupil dynamics, which were beyond the scope of the present study. Future research should address this issue using a multimodal approach that explicitly incorporates hemisphere-specific analyses.

#### 4.2.2 Multimodal response

We discuss why pupil dilation was observed exclusively during the post-stimulus period following the presentation of fear-inducing images. The LC-NA system strongly influences pupil size. In contrast, the enhancement of theta power in brain waves is affected by cortical feedback from the amygdala, while the increase in RRI is driven by vagal activity. Recent studies have shown that LC neurons exhibit two distinct firing modes: tonic discharge, which refers to a constant firing frequency, and phasic discharge, which occurs in response to specific stimuli or events. Under conditions of sustained stress, the LC transitions from a moderate tonic mode to a high tonic mode, and phasic mode activity is suppressed (Janitzky, 2020). Additionally, stress responses have been reported to prolong the activity of the NA system (Valentino and Van Bockstaele, 2008). Based on these findings, the sustained pupil dilation observed during the gray image period following the fear-inducing stimuli may be attributed to an acute stress state induced by the stimuli, which triggered a strong tonic mode in the LC. This sustained dilation likely elevated the baseline pupil size, with effects persisting for a relatively long duration. However, further studies are necessary to validate this hypothesis under experimental conditions that better isolate LC-NA system activity. Such investigations will be essential for a deeper understanding of this phenomenon.

Our findings confirmed that pupil dilation was the most sensitive single indicator of fear, consistent with previous literature on emotional arousal and sympathetic activation (Bradley et al., 2008; Laeng et al., 2012; Mathôt, 2018). While this result does not represent a novel discovery, its replication within a multimodal monitoring context provides empirical support for the robustness of pupillary measures (Mathôt, 2018). Importantly, the study contributes by systematically comparing pupil-based responses with EEG and heart rate measures under controlled conditions, highlighting the practical implications of modality selection for emotional state monitoring.

Regarding additional limitations of this study, although pupil dilation exhibited the highest accuracy in distinguishing which stimulus (fear-induced or neutral) was presented, combining it with EEG and RRI resulted in only limited accuracy improvement (see Figure 4). One possible reason for this limited synergy is that the three modalities may partially reflect overlapping physiological processes related to fear responses, reducing the incremental value of integration. Additionally, differences in temporal dynamics across modalities may have complicated effective integration. From an analytical perspective, our EEG analysis focused primarily on power changes at the sensor level. Importantly, we did not employ source localization or connectivity-based analyses, which could have provided deeper insights into the cortical or large-scale network dynamics associated with fear processing. As EEG and RRI reflect distinct aspects of neural activity beyond autonomic arousal, future studies should consider employing more advanced analytical approaches—such as source-level inference, functional connectivity analyses (ideally using high-density EEG), and complexity measures that capture non-linear dynamics indicative of large-scale neural interactions (Nobukawa et al., 2024)—to better delineate the complementary contributions of these modalities in emotional state monitoring. In addition, heart rate variability (HRV) indices may offer more informative features than simple RRI measures and should be incorporated into future multimodal designs. More limitation of this study is that only male participants were included. This decision was made to control for hormonal fluctuations related to the menstrual cycle, which can affect stress reactivity and emotional processing. Previous studies have shown that hormonal phases can modulate amygdala activity and autonomic responses to emotional stimuli (Andreano and Cahill, 2010). To reduce variability in physiological responses, we chose a homogeneous sample in this initial investigation. However, future studies should incorporate female participants and consider controlling for menstrual cycle phase or hormonal levels to enhance the generalizability and inclusivity of the findings. Furthermore, while we evaluated classification performance using LOOCV, we did not report confidence intervals for the AUC scores. This is primarily due to the small sample size (*N* = 40), which limited the feasibility of reliable resampling-based confidence interval estimation. LOOCV was chosen to maximize training data per fold, a common approach in low-*N* settings, but it inherently lacks variance estimation across folds. Future studies with larger cohorts should adopt cross-validation schemes that allow for statistical resampling (e.g., *k*-fold cross-validation with bootstrapping) to quantify uncertainty and improve the robustness of classification metrics.

#### 4.2.3 Potential applications of multimodal fear detection

Despite the limited accuracy gains from multimodal integration observed in this study, the use of multiple physiological measures remains promising for future applications in emotional state monitoring. While each modality—pupil size, EEG, and heart rate—may partly reflect overlapping processes (Nobukawa et al., 2024), they also capture distinct neurophysiological dimensions of fear, such as sympathetic activation, cortical arousal, and parasympathetic regulation. These complementary features may prove advantageous in more ecologically valid or high-stakes settings where robustness is critical.

Potential applications of such multimodal monitoring include clinical domains (e.g., assessment of preoperative anxiety or PTSD) (Senaratne et al., 2022), or safety-critical environments (e.g., driver monitoring, aviation) (Rastgoo et al., 2018). Furthermore, with advancements in wearable technologies and data fusion algorithms, it may become feasible to implement such systems in real-world contexts, allowing for continuous, adaptive support based on an individual's affective state. Future research should explore how to optimally leverage the complementary features of each modality to improve emotion monitoring in real-world applications.

## 5 Conclusions

In this study, we evaluated psychological and multimodal physiological responses to fear-induced stimuli. The results revealed characteristic responses in each modality, including heart rate, pupil size, and EEG, which likely reflect processes occurring in different neural systems. These findings suggest the potential for future applications in monitoring an individual's fear state by integrating such multimodal neural responses.

## Data Availability

The datasets presented in this article are not readily available because the informed consent did not include the declaration regarding publication of data. Requests to access the datasets should be directed to nobukawa@cs.it-chiba.ac.jp.
